# Bilateral medial medullary infarction with intravenous thrombolytic therapy: A case report

**DOI:** 10.1097/MD.0000000000033375

**Published:** 2023-03-31

**Authors:** Mingyue Fan, Junshu Gao, Na Li, Wei Jin, Yang Liu, Xueqian Zhang, Peiyuan Lv

**Affiliations:** a Department of Neurology, Hebei General Hospital, Shijiazhuang, Hebei, China; b Hebei Provincial Key Laboratory for Cerebral Networks and Cognitive Disorders, Shijiazhuang, Hebei, China.

**Keywords:** bilateral medial medullary infarction, high-resolution magnetic resonance imaging, intravenous thrombolytic therapy, respiratory support, vertebral artery thromboembolism

## Abstract

Bilateral medial medullary infarction is a rare stroke subtype. To investigate its clinical manifestations, etiology, imaging features and thrombolytic effect, We here in reported a patient with acute ischemic stroke in the bilateral medial medullary and reviewed the related literatures.

**Patients concern:**

A 64-year-old female was taken to our hospital after 4.5 hours of dizziness in the morning, followed by somnolence and limb weakness. She gradually worsened into a rapidly progressive tetraparesis and slurred speech.

**Diagnoses:**

Diffusion weighted imaging exhibited a “heart appearance” sign in bilateral medial medulla oblongata, and high-resolution magnetic resonance imaging suggested the left vertebral artery-4 thromboembolism.

**Interventions:**

Timely intravenous thrombolysis was performed.

**Outcome:**

After intravenous thrombolysis, the patient’s symptoms did not worsen in a short time. Although the symptoms were aggravated in the later stage, they were alleviated after active treatment.

**Lessons:**

Diffusion weighted imaging can assist in the early diagnosis of bilateral medial medullary infarction, which will help in the decision to proceed with intravenous thrombolysis therapy. High-resolution magnetic resonance imaging should be improved as soon as possible, which can provide basis for the next intravascular interventional therapy.

## 1. Introduction

Medial medullary infarction is a rare stroke subtype, and bilateral medial medullary infarction (BMMI) is even rarer. Davison first reported a case of BMMI confirmed by autopsy in 1937. The clinical manifestations of BMMI are complex, with large variation in severity and outcome. The early symptoms of the disease are atypical, with a high incidence of respiratory dysfunction. It is worth noting that patients generally have a poor prognosis, often leaving severe neurological deficits, and even death.^[1]^ It is easy to be misdiagnosed and missed in clinical practice, and early treatment is even more difficult to achieve. We here first reported a rare case with BMMI who received intravenous thrombolytic therapy. The clinical data were summarized in this paper.

## 2. Case presentation

Four and a half hours prior to admission, a 64-year-old female suddenly suffered from dizziness in the morning, followed by somnolence and limb weakness. During this period, she gradually worsened into a rapidly progressive tetraparesis and slurred speech. She had a history of hypertension and cerebral infarction. Neurologic examination revealed somnolence, dysarthria, nystagmus, hyporeflexia, flaccid quadriplegia, bilateral Babinski signs without tongue paralysis or sensory disturbance. The power in her bilateral upper limbs was 4 of 5, and in bilateral lower limbs was 2 of 5. Her National Institutes of Health Stroke Scale (NIHSS) score was 14/42.

**Figure 1.**
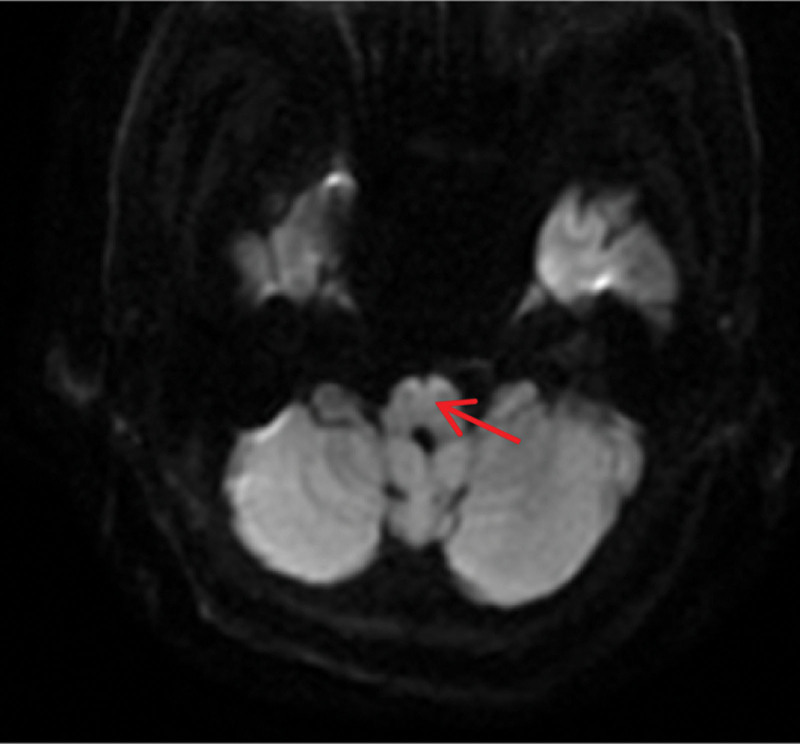
Axial DWI showed a hyperintense “heart appearance” signal in the bilateral anteromedial medulla (red arrow). DWI = diffusion weighted imaging.

**Figure 2.**
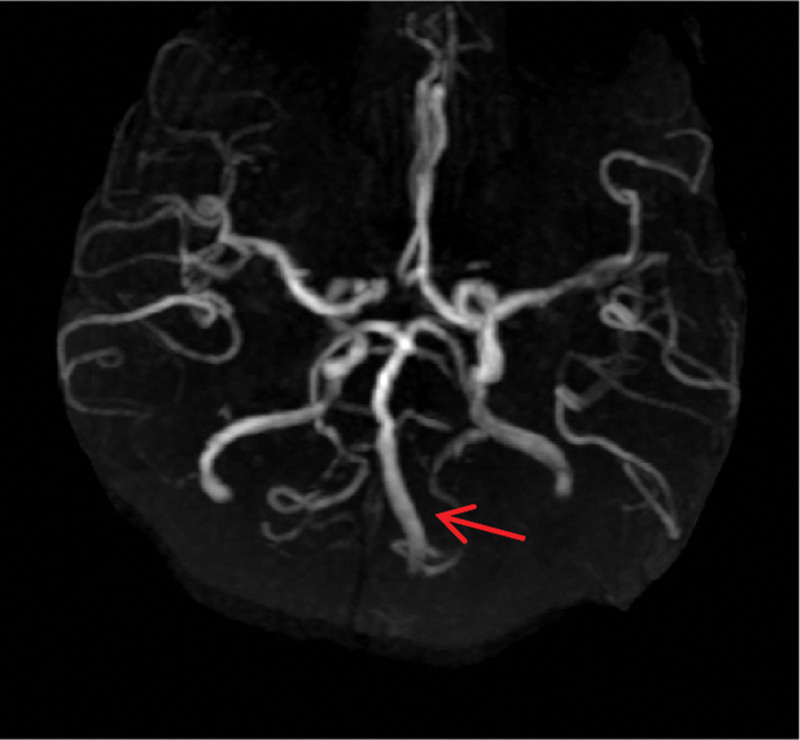
MRA showed the left vertebral artery (VA) occlusion (red arrow).

**Figure 3.**
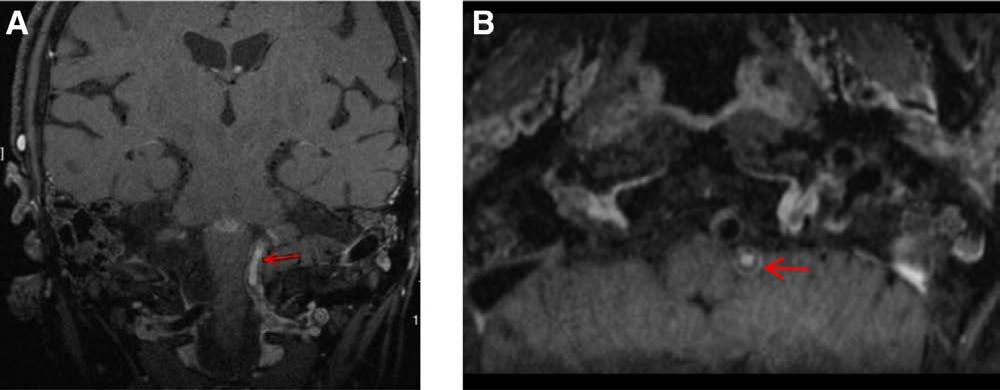
HR-MRI was performed to confirm LV4 thromboembolism (red arrow; (A) coronal view and (B) axial view).

Brain magnetic resonance images obtained 3.5 hours after symptom onset showed a hyperintense “heart appearance” signal in the bilateral anteromedial medulla on diffusion weighted imaging (DWI) imaging (Fig. 1). Magnetic resonance angiography (MRA) showed the left vertebral artery (VA) occlusion (Fig. 2). According to the Chinese guidelines for diagnosis and treatment of ischemic cerebrovascular diseases,^[2]^ the patient was treated with 150W IU urokinase for 30 minutes at about 5 hours after symptom onset. NIHSS score was 14 before and after thrombolytic therapy. There was no aggravation of symptoms after admission. But onset of the 21^st^ hours, the tetraparesis was getting worsen. The muscle power for right limbs was grade 0, and grade 3 for left upper limb and grade 1 for left lower limb. NIHSS score was 18. On the second day of admission, she received an endotracheal intubation due to dyspnea, and a tracheotomy on the 6th day. The power in her 4 limbs improved after 10 days of admission. The ventilator was stopped on day 11, and the tracheal tube was removed on day 21. On the 19th day of hospitalization, high-resolution magnetic resonance imaging (HR-MRI) was performed to confirm LV4 thromboembolism[Fig. 3]. The patient was discharged after 26 days of hospitalization. The muscle strength of the left limb was grade 4, and the right limb was grade 3. She had a modified Rankin Scale score of 2 and NIHSS score of 7 at 3 months after symptom onset.

## 3. Discussion

The anatomy of the medial medulla is complex, which leads to a variety of clinical manifestations. The severity and prognosis of the disease are related to the range and size of infarction. In this study, the patient present weakness of limbs and positive bilateral pathological reflexes, indicating bilateral pyramidal tracts were damaged. Nystagmus was associated with the involvement of the medial longitudinal bundle. Dysphagia is a complex reflex that may be associated with nucleus ambiguus involvement. There was no abnormality in deep and superficial sensibility, suggesting that the medial lemniscus was slightly involved. The lesions are relatively limited, and it is considered to be incomplete related to the ischemic scope in the early stage of cerebral infarction. Dyspnea is another important clinical manifestation of BMMI, with a reported incidence of 24.3%,^[1]^ much higher than the 5% incidence of unilateral medullary infarction.^[3]^ Respiratory dysfunction in medulla oblongata suggested a poor prognosis with a fatality rate of 33%.^[4]^Disappearance of the pharyngeal reflex and palatal palsy on physical examination may warn of dyspnea in patients with bulbar infarction.^[4]^ The location of dyspnea is complicated. Some scholars suggested that: It may be related to the damage of the medulla oblongata respiratory center; It may be related to the involvement of supranuclear fibers dominating the nucleus ambiguus, which causes pseudobulbar palsy and damaging the medullary reticular formation.^[5,6]^BMMI patients mostly presented with complete and severe bulbar paralysis. Meanwhile, the patients are old and frail, resulting in the significantly increased incidence of aspiration pneumonia and hypostatic pneumonia. This may also be a cause of respiratory failure and a major cause of death. On admission, the patient had dysarthria and pharyngeal reflex dullness, indicating the presence of pseudobulbous paralysis. On the second day of admission, the patient developed cough and difficulty in expectoration, suggesting dyspnea. She received an endotracheal intubation because of dyspnea, and a tracheotomy mask on day 6 of hospitalization. This successfully helped the patient overcome breathing difficulties. The ventilator was stopped on day 11, and the tracheal tube was removed on day 21.

The medulla oblongata has a rich and unique vascular network. According to vascular innervation, the medulla oblongata can be divided into the anteromedial area, anterolateral area, lateral area and dorsal area in axial position. The affected areas in BMMI are mainly anteromedial area and anterolateral area. The arteries dominating these areas are vertebral artery and anterior spinal artery.^[7]^ The anteromedial region is mainly dominated by the vertebral artery at the upper part of the medulla oblongata, and by the anterior spinal artery at the lower part of the medulla oblongata. While the anterolateral region is mainly dominated by the anterior spinal artery.^[8]^Pongmoragot et al^[1]^ conducted a retrospective analysis of the MRA of 38 patients with BMMI and found that BMMI might be related to artery stenosis or occlusion, including VA atherosclerosis (38.5%), VA occlusion (15.4%), basilar artery therosclerosis (19.2%), dissection (7.7%), anterior spinal artery occlusion

(3.8%; an autopsy case), and no abnormalities (38.5%). These findings suggest that aorta atherosclerosis, especially vertebrobasilar artery stenosis/occlusion, is the main vascular imaging basis of BMMI. In some patients, no significant stenosis/occlusion was found and atherosclerotic plaque was presumed to block the vertebrobasilar perforator opening. It may be a contralateral thrombosis at the vertebrobasilar junction or a variation in the perforating vessels supplying the medial medulla oblongata of both sides, that is, 1 vertebral artery supplying the medial medulla oblongata of both sides.^[9,10]^Hypertension, diabetes, smoking and dyslipidemia are important causes of atherosclerosis. Conventional angiography such as MRA and computer tomography angiography is more accurate to assess intracranial vascular lumen status, but its specificity and sensitivity are not high. At the same time, it cannot reflect the morphological and structural characteristics of arterial wall and atherosclerotic plaque, which is not conducive to accurate diagnosis. In recent years, the application of HR-MRI has gradually become popular in clinical practice. Besides, it can objectively and accurately display the structure of arterial wall and help to diagnose atherosclerotic stenosis. It can also accurately reflect the morphological and structural characteristics of micro atherosclerotic plaques and be used to identify types of posterior circulation ischemia. At the same time, it is a noninvasive test and there is no radiation damage. In this case, the patient had atherosclerotic risk factors such as hypertension and diabetes history. HR-MRI confirmed thromboembolism in the V4 segment of the left vertebral artery (VA). It was speculated that the disease was caused by atherosclerosis. The underlying mechanism was that bilateral anterior spinal artery originated from ipsilateral vertebral artery, and BMMI can occur on the basis of arteriosclerosis with unilateral VA occlusion.

Due to the diversity of range of ischemia, the speed of disease progression and individual differences, the clinical manifestations of bilateral bulbar infarction are complex and diverse. It is difficult to diagnose at the early stage only according to clinical symptoms. MRI examination can clearly show the brain stem structure, and DWI can show high signal in the lesion area at ultra-early stage. Therefore, its early diagnosis has recently become possible by DWI sequence, which shows the characteristic “heart appearance” sign.^[11]^ To the best of our knowledge, early intravenous thrombolytic therapy is an effective way to improve the prognosis of patients with acute cerebral infarction. However, it is a pity that there is no report of intravenous thrombolytic therapy for BMMI. The reasons for patients not receiving thrombolytic therapy were analyzed to be related to delayed treatment, failure to diagnose in time and missing the time window of thrombolytic therapy. In addition, even within the time window, due to the complex and diverse clinical symptoms, the basic clinicians do not have enough understanding of the disease, which is also related to the failure of timely thrombolysis. In this report, high signal in bilateral medial medulla oblongata was clearly identified by DWI in the patient admitting, which provided image support for the diagnosis of acute cerebral infarction. Combined with symptoms and signs, BMMI was taken into account. According to Chinese Guidelines for the Diagnosis and Treatment of Acute ischemic Stroke 2018,^[2]^ 150W IU urokinase was given for 30 minutes at about 5 hours after onset. NIHSS score was 14 before thrombolytic therapy and still 14 afterward. The patient’s symptoms did not get worse, therefore, it was considered thrombolytic therapy was successful in restoration of neurological function.

According to pervious publications, most patients with bilateral medial medullary infarction subject to poor prognosis. The prognosis depends on the speed, location, and size of the thrombus, the extent of cerebral edema, the presence or absence of vascular occlusive disease elsewhere in the posterior circulation, and collateral circulation. Acute respiratory failure and quadriplegic muscle strength to grade 0 strongly suggest poor prognosis.^[12]^ Pongmoragot et al^[1]^ reported that fatality rate during hospitalization was 23.8%, and 61.9% of patients needed other people to take care of their daily life for 2 to 3 months after onset. There are also case reports with better prognosis. In particular, with the application of DWI in recent years, the early diagnosis and reasonable treatment of BMMI have enabled, and the cases with good prognosis have gradually increased. Due to timely intravenous thrombolysis, the patient’s symptoms did not worsen in a short period of time. Although the symptoms worsened later, the patient was actively treated with aspirin and atorvastatin, as well as ventilater-assisted breathing, and the symptoms were relieved at 26 days after discharge. The muscle strength of the left limb was grade 4, and the right limb was grade 3. She had a modified Rankin Scale score of 2 and NIHSS score of 7 at 3 months after symptom onset. The good recovery of the patient was related to the fact that the infarct was relatively small and did not involve the lateral and dorsal areas. In addition, timely intravenous thrombolytic therapy for the patient did not lead to further enlargement of the infarction focus, and early timely endotracheal intubation to avoid respiratory dysfunction also helped improve the prognosis of the patient. As for the thrombolytic effect in BMMI, more cases reports will be needed for further research.

In summary, the clinical manifestations of this disease are complex, with atypical early symptoms and a high incidence of respiratory dysfunction. Simple CT examination is prone to misdiagnosis and missed diagnosis. Therefore, it is necessary to conduct further MRI examination, especially DWI sequence. Since it can show high signal in lesion area in super early stage of infarction. For further etiological diagnosis, computer tomography angiography or MRA examination is feasible to identify the damaged artery. The recent development of HR-MRI also provides a new method for vascular evaluation. Furthermore, the disease usually has a poor prognosis, with outcomes related to the rate of disease progression, infarct location, size, and collateral circulation. Early diagnosis and intravenous thrombolytic therapy may improve the prognosis of patients.

## Author contributions

Conceptualization: Mingyue Fan, Peiyuan Lyu.

Formal analysis: Mingyue Fan, Peiyuan Lyu.

Writing – original draft: Mingyue Fan.

Writing – review & editing: Junshu Gao, Na Li, Wei Jin, Yang Liu, Xueqian Zhang.
